# *Drosophila* pan-glial inducible Gal4 line alters baseline sleep

**DOI:** 10.17912/micropub.biology.002180

**Published:** 2026-05-15

**Authors:** Seanna E Kelly, Keisuke Fukumura, Daridh Mao, Vinithra Kathirvel, Shorbon Mowla, Annika F Barber

**Affiliations:** 1 Waksman Institute, Rutgers, New Brunswick, NJ, US

## Abstract

The GeneSwitch system (GS) is a genetic reagent for pharmacologically inducible gene expression in *Drosophila* using the Gal4-UAS binary expression system. We have identified that the pan-glial *repo* -GS-Gal4 line uniquely causes significant baseline sleep reduction during both day and night in the presence of the inducing agent RU-486 even in the absence of effector transgene expression. Careful experimental design to permit detection of small sleep changes that may be obscured by the large sleep reduction in the presence of RU-486 is required when using the *repo* -GS-Gal4 line for behavioral studies.

**Figure 1. repo -GeneSwitch-Gal4 substantially reduces baseline sleep at all concentrations of RU-486:** (A) Quantification of 24-h sleep time over 7 days for ; *repo* -GeneSwitch-Gal4/+; flies fed 0 (vehicle control), 50, 100, 200, or 465 µM concentration of RU-486 food. Asterisks denote significant difference in sleep time from vehicle control. (B) Relative *gfp* mRNA expression normalized to *a-tub* in heads of ; *repo* -GeneSwitch-Gal4/UAS-nls.GFP; flies fed 0 (vehicle control), 25, 50, 100, 200, or 465 µM RU-486. Asterisks denote significant difference GFP expression from vehicle control, there were no statistically significant differences between drug-fed groups. (C) As in (A) for ;; *nSyb* -GeneSwitch-Gal4/+ flies. Asterisks denote significant difference in sleep time from vehicle control. (D) As in (B) for ;UAS-nls.GFP/+; *nSyb* -GeneSwitch-Gal4/+ flies. Asterisks denote significant differences for the indicated comparisons.

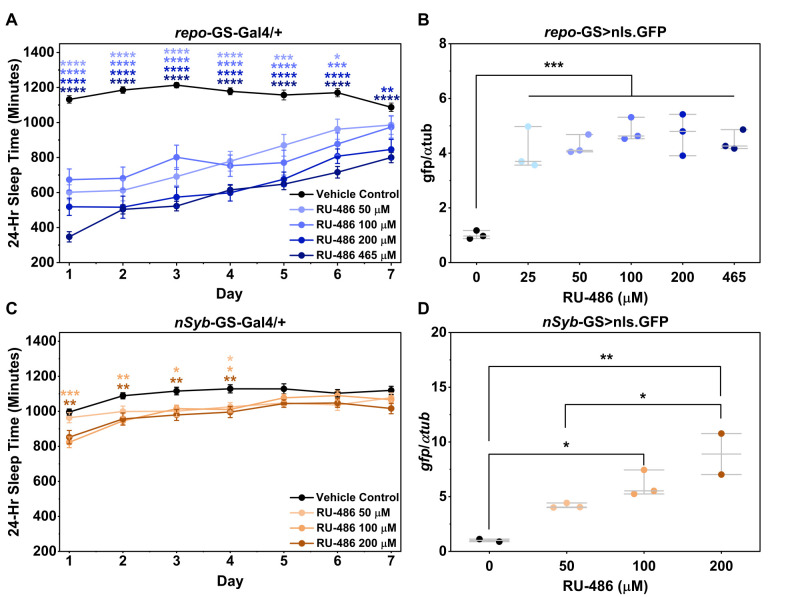



## Description

Inducible gene expression tools in *Drosophila* are widely used within the fly community for temporal control of gene expression. One such system is GeneSwitch (GS), an inducible Gal4 system in which Gal4 is inactive under baseline conditions and activated upon feeding the progesterone antagonist RU-486 (Osterwalder et al., 2001) [citation]. Within the fly sleep field, GS-Gal4s have been used to induce adult-specific changes in gene expression followed by evaluation of changes in sleep amount and sleep architecture to determine ongoing adult roles for genes regulating sleep (Artiushin et al., 2018; Axelrod et al., 2023; Cho et al., 2026; Han et al., 2024; Joiner et al., 2006; Kuo et al., 2010; F. Li et al., 2023; Q. Li & Stavropoulos, 2016; Y. Li et al., 2023, 2024; Ly et al., 2020; Pyfrom et al., 2025; Tabuchi et al., 2015; Wu et al., 2009). Gal4-GS expression or RU-486 feeding alone do not affect sleep behavior in adult flies, although RU-486 exhibits developmental toxicity (Afonso et al., 2015; Q. Li & Stavropoulos, 2016; Wu et al., 2009). Several studies have noted that *repo* -GS-Gal4 flies have reduced sleep compared to UAS control lines when fed RU-486 (F. Li et al., 2023; Y. Li et al., 2023; Pyfrom et al., 2025). Similar sleep reductions have also been noted using the *MB* -GS-Gal4 (mushroom body) driver, but not the *nSyb* -GS-Gal4 (pan-neuronal) and *da* -GS-Gal4 (ubiquitous) drivers (Artiushin et al., 2018; Cho et al., 2026; Y. Li et al., 2023; Ly et al., 2020; Tabuchi et al., 2015; Wu et al., 2009). Concentrations of RU-486 used for sleep studies vary widely, from 100 µM to 5 mM, yet sleep deficits occurred in *repo* -GS-Gal4 and *MB* -GS-Gal4 flies across concentrations and time spent on RU-486 (Artiushin et al., 2018; Axelrod et al., 2023; Joiner et al., 2006; Pyfrom et al., 2025; Wu et al., 2009).

Just two prior studies have compared sleep metrics of *repo* -GS-Gal4 in the presence and absence of RU-486 (Cho et al., 2026; Pyfrom et al., 2025). Cho et al. (2026) find ~100 minutes of sleep loss per day for *repo* -GS-Gal4 flies on 0.5 mM RU-486, though the profile of sleep loss over time on RU-486 is not shown. Pyfrom et al. (2025) show average 24 h sleep profiles identifying substantial day- and nighttime sleep loss for *repo* -GS-Gal4 in the presence of 100 µM RU-486 compared to vehicle control. We sought to identify an RU-486 concentration at which *repo* -GS-Gal4 would have minimal sleep loss, while still inducing gene expression. We found that *repo* -GS-Gal4 has significantly and substantially reduced sleep at all RU-486 concentrations, a property unique to this GS line among those previously tested in the literature. The strong reduction in baseline sleep in *repo* -GS-Gal4 flies in the presence of RU-486 raises concerns around the utility of the system for genetic screens of sleep modulation.

We characterized baseline sleep behavior and transgene expression levels using the pan-glial *repo* -GS-Gal4 and pan-neuronal *nSyb* -GS-Gal4 lines at multiple RU-486 concentrations. Using either *repo* -GS-Gal4 or *nSyb* -GS-Gal4, we measured baseline sleep with the *Drosophila* activity monitoring (DAM) assay and evaluated UAS-transgene expression levels by q-PCR at varying RU-486 concentrations. Both GS lines were backcrossed to our *w* ^1118^ isogenic line (Iso ^31^ ) (Ryder et al., 2004) for at least 6 generations to control for genetic background. Flies were placed on either ethanol vehicle control, 25, 50, 100, 200, or 465 µM concentrations of RU-486 mixed into agar/sucrose food for 24-hours prior to sleep experiments or into Bloomington fly diet for qPCR analysis one week prior to assay.

Upon examining baseline sleep over 7 days, we found that RU-486 significantly and substantially reduced sleep in *repo* -GS-Gal4 flies. 24-hour sleep time was significantly reduced at all concentrations of RU-486 for *repo* -GS flies compared to vehicle control ( Figure 1A ). Baseline 24-h sleep of *repo* -GS flies on vehicle control had a 7-day average of 1140 ± 9 minutes and average sleep time decreased dose-dependently with RU-486 concentration: 300 ± 23 minutes loss at 50 and 100 µM, 400 ± 23 minutes loss at 200 µM, and 550 ± 14 minutes loss at 465 µM. As in other studies, we found that RU-486-induced sleep loss in *repo* -GS-Gal4 flies was larger in the day (zeitgeber time 0-12) than at night (zeitgeber time 12-24), however sleep loss was significant during both periods. During the day, flies lost ~300 minutes of sleep at all RU-486 concentrations, while at night sleep loss was more concentration dependent, with ~300 minutes of sleep loss at 200 µM RU-486 but only ~150 minutes at 50 or 100 µM RU-486. We noted that the effect of RU-486 feeding on sleep loss is ameliorated over time in the assay. Despite this amelioration, by day 7 in the assay, flies fed 200-465 µM RU-486 still lost significantly more sleep than vehicle controls.

These data suggest that sleep quantity in *repo* -GS-Gal4 flies is highly sensitive to RU-486 feeding even in the absence of transgene expression. qPCR analysis of *repo* -GS-Gal4-driven UAS-nls.GFP expression after 24 h of RU-486 feeding revealed that the *repo* -GS system is highly sensitive to RU-486 induction. All RU-486 concentrations tested resulted in maximal GFP in fly heads, with no significant difference in GFP expression from 25-465 µM RU-486 ( Figure 1B ). Our qPCR findings indicate that the *repo* -GS system is highly sensitive to even very low levels of RU-486 feeding for just 24 hours.

The large sleep reducing effects of RU-486 do not extend across all GS-Gal4 lines. *nSyb* -GS flies still displayed a significant sleep reduction in response to RU-486 feeding on the first four days in the assay ( Figure 1C ). However, the absolute amount of sleep lost in *nSyb* -GS flies is only ~200 minutes/day over each of the first four days at all RU-486 concentrations tested. Baseline 24-hr sleep of *nSyb* -GS flies on the vehicle control averages 1097 minutes over 7 days in the assay and does not show dose dependent decreases with RU-486 concentration: 100-minute sleep loss (± 9-12 minutes) for 50, 100, and 200 µM. Although RU-486 induces significant sleep loss in *nSyb* -GS flies over the first few days in the DAM assay, the absolute amount of sleep lost is never more than 200 minutes per day, compared to the loss of 500-800 minutes per day in *repo* -GS-Gal4 flies. Sleep loss in *nSyb* -GS flies is also time-of-day dependent, with more sleep loss at night than during the day at all RU-486 concentrations tested. UAS-nls.GFP induction in heads by *nSyb* -GS-Gal4 is dose dependent with at least 100 µM RU-486 required for a significant induction compared to vehicle control ( Figure 1D ).

Our findings highlight that *repo* -GS-Gal4 is uniquely sensitive to RU-486-induced sleep reduction in the absence of transgene induction. Previous studies found that *repo* -GS-Gal4 is leaky, with *repo* -GS-driven expression of green fluorescent protein (GFP) detected in some glial subtypes in the absence of RU-486 feeding (Artiushin et al., 2018). This leakiness is unlikely to underlie observed sleep reduction in the presence of *repo* -GS-Gal4 with RU-486, as there is no UAS-transgene available to express. The effect of *repo* -GS-Gal4 could be positional, as it is an insertion of a fragment of the glial-specific *reversed polarity* transcription factor gene inserted into the AttP40 docking site on chromosome II while *nSyb* -GS-Gal4 is inserted into the AttP2 site on Chromosome III. Regardless of the underlying cause, using *repo* -GS to assess sleep changes requires careful use of both genetic and pharmacological controls to ensure that sleep changes can be detected at the concentration of RU-486 used. In some cases, the large sleep reductions induced by RU-486 feeding alone may obscure small differences in sleep induced by transgene expression. Alternatives to the GS system should be used to validate sleep findings using the *repo* -GS system, such as the temperature sensitive Gal80 or Q systems. Previous studies in *Drosophila* have successfully used these inducible gene expression approaches to analyze sleep behavior (Q. Li & Stavropoulos, 2016; Y. Li et al., 2023). Other studies have used the GS system to measure debris clearance, axotomy, short- and long-term memory, and synaptic remodeling (Chang et al., 2025; Dissel et al., 2015; Jacobs & Sehgal, 2020; Szabó et al., 2023; Vincze et al., 2026). Given our observations of the effect of *repo* -GS on sleep, careful use of controls is warranted for any phenotype, as short sleep could indirectly affect other outcomes.

## Methods

***Fly Husbandry***

Fly stocks used were *repo* -GS-Gal4 (BDSC # 95307), *nSyb* -GS-Gal4 (Bedont et al., 2021), UAS-nls.GFP (BDSC # 4776), and Iso ^31^ (BDSC #5905). Geneswitch-Gal4 lines were back-crossed to Iso31 for 6 generations to normalize genetic background. All stocks were maintained on Bloomington Drosophila Stock Center cornmeal food in a 12:12 light-dark cycle at 25° C.

***Drosophila Activity Monitor (DAM) Assay***

*repo* -GS-Gal4 and *nSyb* -GS-Gal4 virgins were collected and crossed to Iso ^31^ males. Male ; *repo* -GS-gal4/+; and ;; *nSyb* -GS-Gal4/+ progeny were collected 2-3 days post-eclosion and were placed in vials containing RU-486 DAM food (5% sucrose and 2% agar) or DAM food with 80% ethanol vehicle. Flies were placed on RU-486 food for 48 hours prior to being loaded into the DAM assay. Flies were placed in individual DAM tubes containing RU-486 or vehicle DAM food in one end and a cotton plug in the other. Flies were monitored in the assay for 7 days in a 12:12 light-dark cycle at 25° C. Sleep analysis was conducted using PHASE (Persons et al., 2022) and one-way ANOVA with Tukey’s *post hoc* test was used to determine statistical significance.

***RU-486 Stock Dilutions***

RU-486 is from Tokyo Chemical Industry (TCI), catalogue # TCI-M1732-1G. RU-486 was resuspended in molecular grade ethanol (80%) for a stock solution of 20 mg/mL. Working solution was further diluted to 20 mg/mL in 80% ethanol. All solutions were stored at -20° C.

***Fly head qPCR***

Total RNA was extracted from 50 heads per biological replicate, collected from 10-11-day old adult males maintained on RU-486 or vehicle diet for 7 days prior to sample collection. Tissues were homogenized in TRIzol™ Reagent (Invitrogen, Carlsbad, CA) and purified using the PureLink RNA Micro Kit (Invitrogen) with on-column PureLink DNase treatment according to the manufacturer's protocol. 100 ng of RNA was reverse transcribed using SuperScript IV Reverse Transcriptase (Invitrogen). Quantitative RT-PCR was performed on a CFX Opus 384 Real-Time PCR System (Bio-Rad, Hercules, CA) using iTaq™ Universal SYBR® Green Supermix (Bio-Rad). Cycling conditions were 95° C for 3 m, followed by 40 cycles of 95° C for 10 s and 60° C for 30 s, followed by melt curve analysis. Each sample was run in 3 technical replicates across 2–3 biological replicates. Relative expression levels were calculated by the DDCt method and normalized to *a-tubulin* . Sequences of primers are as follows: *a-tubulin* -Fw 5'-CGTCTGGACCACAAGTTCGA-3', *a-tubulin* -Rv 5'-CCTCCATACCCTCACCAACGT-3', *gfp* -Fw 5'-ATTGGCGATGGCCCTGTCCT-3', *gfp* -Rv 5'-GTTCATCCATGCCATGTGTAATCC-3'.
